# Metrical Presentation Boosts Implicit Learning of Artificial Grammar

**DOI:** 10.1371/journal.pone.0112233

**Published:** 2014-11-05

**Authors:** Tatiana Selchenkova, Clément François, Daniele Schön, Alexandra Corneyllie, Fabien Perrin, Barbara Tillmann

**Affiliations:** 1 Lyon Neuroscience Research Center, Auditory Cognition and Psychoacoustics Team, Centre National de la Recherche Scientifique, Unité Mixte de Recherche 5292, Institut National de la Santé et de la Recherche Médicale, Unité 1028, University Lyon 1, Lyon, France; 2 Cognition and Brain Plasticity Group, Bellvitge Biomedical Research Institute, L’Hospitalet de Llobregat (Barcelona), Barcelona, Spain; 3 Department of Basic Psychology, University of Barcelona, Barcelona, Spain; 4 Attention, Perception and Acquisition of Language Lab, Hospital Sant Joan de Déu, Barcelona, Spain; 5 Aix-Marseille Université, Institut de Neurosciences des Systèmes, Marseille, France; 6 Institut National de la Santé et de la Recherche Médicale, Unité 1106, Marseille, France; University of Texas at San Antonio, United States of America

## Abstract

The present study investigated whether a temporal hierarchical structure favors implicit learning. An artificial pitch grammar implemented with a set of tones was presented in two different temporal contexts, notably with either a strongly metrical structure or an isochronous structure. According to the Dynamic Attending Theory, external temporal regularities can entrain internal oscillators that guide attention over time, allowing for temporal expectations that influence perception of future events. Based on this framework, it was hypothesized that the metrical structure provides a benefit for artificial grammar learning in comparison to an isochronous presentation. Our study combined behavioral and event-related potential measurements. Behavioral results demonstrated similar learning in both participant groups. By contrast, analyses of event-related potentials showed a larger P300 component and an earlier N2 component for the strongly metrical group during the exposure phase and the test phase, respectively. These findings suggests that the temporal expectations in the strongly metrical condition helped listeners to better process the pitch dimension, leading to improved learning of the artificial grammar.

## Introduction

Humans develop expectations about regularities occurring in structures encountered in everyday life (e.g., language, music). Expectations about future events facilitate the processing of expected events in comparison to unexpected events. Humans can learn structural regularities implicitly by mere exposure, that is, in an unconscious way. Implicit learning (IL) is the acquisition of knowledge without the intention to learn [1], [2]. Three main paradigms to study IL are the serial reaction time task (SRTT) [3], artificial language learning (ALL) [4], [5] and artificial grammar learning (AGL) [2]. Of interest here is the AGL paradigm. Participants are first exposed to grammatical sequences, and then, in the test phase they successfully provide grammaticality judgments that distinguish between new grammatical sequences and ungrammatical sequences without being able to explain their choice, suggesting implicit grammar knowledge acquired during the exposure phase.

In addition to behavioral measures (i.e. number of correct responses and response times) in IL experiments, neurophysiological methods, such as electroencephalography (EEG), functional magnetic resonance imaging and transcranial magnetic stimulation have been used to investigate neural correlates underlying IL (e.g., [6]–[10]). For example, the IL studies using EEG have shown that violations to newly learned sequence structures alter event-related potential (ERP) components, such as the N1, N2, N2/P3 complex, P300, N400, P600 components (e.g., [6], [7], [11]–[15]). Using the AGL paradigm, Carrión and Bly [6] demonstrated that ill-formed test sequences elicited a larger N2b/P3 complex (time-locked to the event that violates the grammar) in comparison to well-formed sequences. The N2/P3 complex has been also shown in studies that compared IL and explicit learning [12], [16], [17]. These studies suggested that the N2/P3 complex arises when explicit knowledge was acquired. However, when participants were unaware of the sequential presentation (i.e. implicit knowledge), only the N2 component showed the difference between well-formed and ill-formed sequences.

While most AGL studies presented the events of the artificial sequences simultaneously in the visual modality, some studies used a sequential presentation in the auditory modality, keeping the temporal presentation of the material constant. However, it is well known that the temporal structure of event presentation can influence auditory processing, notably leading to facilitated event processing when presented in isochronous patterns or regular temporal patterns in comparison to irregular temporal patterns [18]–[23]. Temporal expectations appear to facilitate not only auditory event perception (see above), but also event discrimination [18], [19] and implicit learning [24], [25].

The influence of temporal presentation on auditory processing may be explained in the framework of the Dynamic Attending Theory (DAT) [26], [27]. The DAT postulates that external temporal regularities between events can entrain internal oscillators that guide attention over time and help listeners to develop temporal expectations for future events. In line with the DAT, Schmidt-Kassow et al. [22] showed that the temporal predictability of the occurrence of a deviant stimulus leads to larger amplitude and shorter latency of the P3b than deviants in temporally random sequences. Schwartze et al. [23] further compared the influence of isochronous and random presentations of tone sequences on ERPs related to an oddball paradigm in pre-attentive (participants did not pay attention to the stimuli) and attentive conditions (participants performed a task related to the tone sequences). As in Schmidt-Kassow et al. [22], Schwartze et al. [23] found that deviant tone processing elicited a larger P3b in the isochronous presentation than in the random presentation in the attentive condition, but no influence of temporal structure was found in the pre-attentive condition.

According to the DAT and the metric binding hypothesis [28], meter may modulate attentional resources over time and thus influence temporal expectations for future events. Meter can be conceived as a cognitive construct that has a hierarchical structure with stressed and unstressed elements superimposed on an underlying regular pulse. Selchenkova et al. [25] investigated whether temporal regularities influence the learning of pitch structures based on an artificial grammar. Two different sets of temporal patterns, i.e. regular and irregular, were created to be associated with artificial grammar of tone sequences in the exposure phase. Regular patterns were constructed to allow for the abstraction of a clear metrical framework. In contrast, irregular patterns were constructed by reorganizing the same temporal intervals to disrupt this metrical framework. This study revealed that the learning of the artificial grammar was facilitated by the regular presentation (i.e. strongly metrical) of the tone sequences in the exposure phase in comparison to the irregular presentation. Interestingly, results in the strongly metrical condition were highly similar to the results of Tillmann and Poulin-Charronnat [29], wherein the same artificial grammar was presented isochronously. However, on the basis of the DAT and its metrical binding hypothesis [28], one would expect a greater attentional peak at expected points in the strongly metrical presentation, leading to a more pronounced benefit compared to the isochronous presentation.

Our present study aimed to compare directly the IL of an artificial pitch grammar presented with two different regular temporal structures. The tone sequences were associated either with strongly metrical patterns or with an isochronous pattern and presented each to a different group of participants. Combining behavioral and ERP measurements allows studying whether a metrical framework may provide an additional benefit for learning in comparison to an isochronous presentation. In line with other studies on implicit learning (e.g., [6]), the difference in amplitude and/or latency of ERP components between ungrammatical target events versus grammatical target events might be an indicator of perceived grammatical violations and thus a marker of the acquired grammar knowledge. We made the hypothesis that if the strongly metrical presentation in the exposure phase helps developing stronger temporal expectations than the isochronous presentation, this should lead to enhanced grammar learning, as revealed in the test phase: correct responses should be more numerous and/or ERP differences between ungrammatical and grammatical target tones should be larger and/or start earlier for the participant group with the strongly metrical presentation than for the group with the isochronous presentation.

In addition, the advantage for event processing thanks to the metrical structures may also be reflected in the exposure phase. To keep participants attentive, we introduced some mistuned tones in the exposure phase and participants had to detect them. We hypothesized that the strongly metrical presentation should facilitate pitch processing and thus facilitate mistuned-tone processing in comparison to an isochronous presentation.

## Materials and Methods

### Participants and ethics statement

Thirty-one university students participated in this experiment (10 men). Five participants were discarded from EEG analyses due to major artifacts, resulting in two groups of 13 participants. Age ranged from 21 to 29 years, with a mean of 23.54 (*SD* = 2.44). Participants were randomly assigned to one of the two experimental groups listening either to the isochronous material (referred to as the Iso group hereafter) or the strongly metrical material (referred to as the SM group hereafter). All participants signed informed consent before the experiment. The present experimental paradigm and the written informed consent were approved by the French ethics committee, Comité de Protection de Personnes Sud-Est II. After the experiment, they were asked to fill out a questionnaire about musical experience (including years of dancing, singing, playing musical instruments (if any) and a self estimation of their sense of rhythm on a 5-point scale where “1” = “I don’t have any sense of rhythm” and “5” = “yes, I have very good sense of rhythm”). One additional participant in the Iso group was discarded from the analyses of the exposure phase due to major EEG artifacts.

The two groups of participants did not differ in their musical, dancing and singing experience (musical experience: 0.85 years (*SD* = 1.28) for the Iso group, 0.40 years (*SD* = 1.38) for the SM group, *p* = .40; dancing experience: 3.46 years (*SD* = 1.19) for the Iso group and 2.69 years (*SD* = 1.32) for the SM group, *p* = .13; singing experience: 1.31 years (*SD* = 3.09) for the Iso group and 1.15 years (*SD* = 2.82) for the SM group, *p* = .89). The groups also did not differ in their self-reported sense of rhythm (2.85 (*SD* = 0.89) for the Iso group and 3.0 (*SD* = 0.82) for the SM group, *p* = .46). Participants had self-reported normal hearing and none of them reported to have absolute pitch.

### Stimuli

#### Exposure phase

The material was based on the artificial grammar from Tillmann and Poulin-Charronnat [29], which was adapted from previously used grammars (e.g., [30]). For this finite-state grammar, five pitches were used: a3, a#3, c4, d4, f#4.

For the exposure phase, thirteen 10- and twenty-two 12-tone grammatical sequences (thirty-five grammatical sequences in total) were coupled to fourteen different strongly metrical patterns in such way that each grammatical sequence was associated with four different 10-item and 12-item strongly metrical patterns. In the isochronous condition, the thirty-five 10- and 12-tone grammatical sequences were all coupled to an isochronous pattern. This allowed us to create 140 sequences for the strongly metrical exposure phase and 140 sequences for the isochronous exposure phase.

Each tone lasted 220 ms. The inter-onset-intervals (IOIs) were 220, 440, 660 or 880 ms for the strongly metrical patterns and 570 ms for the isochronous pattern. Thus, the sequences in both temporal conditions had similar durations: the 10- and 12-tone exposure sequences with strongly metrical presentation lasted for 5,500 and 6,380 ms and the 10- and 12-tone sequences with the isochronous presentation lasted for 5,350 and 6,490 ms, respectively.

During the exposure phase, one mistuned tone (-52 cents) was inserted in 25% of the 140 sequences. The serial position of this mistuned tone varied across sequences from the second to the ninth tone position.

The strongly metrical patterns (taken from [25]) were constructed to ensure the establishment of a strong metrical framework. For this, IOIs were organized to respect oscillatory cycles at two levels, i.e. with a period of 440 ms and a period of 880 ms. The temporal structure of each temporal pattern had been analyzed at global and local levels. Global metric structure was assessed using two indices provided by Povel and Essens’s Clock model [31] (a C-score indicating the best internal clock for the smallest obtained values) and the DAT theory (a DAT index; developed in [25] based on [26]), respectively. For the Clock model, a C-score of 0, which thus indicates strongest metric regularity, was found for all strongly metrical patterns. The DAT index reflects the difference between the number of accented and the number of unaccented tones in a sequence. Calculating this index for our patterns also confirmed high metric regularity of the strongly metrical patterns. Local temporal structure features (e.g., rhythmic groupings and frequency analyses of IOI co-occurrences) demonstrated low variability and relatively high repetitions of IOI pairs (local temporal structure within three successive tones) and IOI triplets (local temporal structure within four successive tones), thus further highlightening the temporal regularity of the strongly metrical patterns. In sum, global and local metric analyses confirmed the metrical characteristics of the patterns (see [25] for analyses and more details about patterns construction). Audio examples of the exposure and test sequences (Sound examples S1–S5) and temporal patterns (Table S1) are available in Supporting information.

#### Test phase

The test phase comprised thirty-six new grammatical sequences and thirty-six ungrammatical sequences. The ungrammatical test sequences differed by one tone from the grammatical sequences. This tone was part of the finite-state grammar, but created controlled subtle grammatical violations; for example, the ungrammatical tone did not create new bigrams (see [29] for more details). Sequences were played with either strongly metrical patterns for the SM group or isochronously for the Iso group. In all sequences, each tone lasted 220 ms.

### Procedure and Equipment

For the present study, we have developed an adaptation of previously used implicit learning paradigms that aimed to avoid informing participants about the rule-governed nature of the grammatical melodies during the entire experiment (exposure phase and test phase) and thus allowing for a more direct investigation of implicit learning processes (see also [29]).

In the exposure phase, participants were told that a beginner pianist played all melodies and that after recording we randomly introduced a mistuned tone. They were asked to indicate whether each sequence contained a mistuned tone or not. This mistuned tone-detection task aimed to keep participants’ attention to the stimuli during the exposure phase, and in particular to the pitch dimension of the stimuli (that is, the dimension of the to-be-learned grammatical structures). This task was adapted from [29] (Experiment 2).

During the test phase, the sequences were presented by pairs. Each pair contained one new grammatical sequence and one ungrammatical sequence (order was counterbalanced). Participants were asked to indicate which melody was played by the same pianist than the one of the exposure phase. With this instruction, we made the hypothesis (even though we did not explicitly say so) that the pianist beginner was playing melodies of a specific style or repertoire. After having given this judgment, participants rated the confidence of their decision on a 5-point Likert scale (where “1” was not confident at all, “5” was highly confident and “0” was guessing). The test phase contained 72 trials presented in random order for each participant and in two blocks with a small break between them.

The experiment was run using the software Presentation (Neurobehavioural Systems). Stimuli were delivered binaurally using air-delivery headphones (ER-2, Etymotic).

### Data acquisition and analyses

#### Behavioral analyses

For the exposure phase, correct responses for mistuned tones (Hits) and false alarms (mistuned tone responses for in-tune sequences) were calculated for each participant. For the test phase, correct responses for grammatical and ungrammatical target tones were calculated for each participant. The one-sample Wilcoxon signed rank test was used to test whether participants’ performance was above chance, and the Mann-Whitney U test was used to compare groups’ performance. To obtain an index of explicit vs. implicit acquired knowledge, confidence ratings were calculated separately for correct and incorrect responses, and analyzed with a 2×2 ANOVA with responses (correct/incorrect) as within-participant factor and group (SM, Iso) as between-participants factor. Difference between confidence ratings to correct and incorrect responses suggests explicit knowledge, whereas no difference suggests that learning was implicit. Note that participants were not informed about grammatical rules of the sequences and this index thus indicated knowledge related to the cover story of the pianist.

#### ERP acquisition and analyses

Participants were comfortably seated in a sound-attenuated booth in front of a monitor. The EEG signal was recorded from 95 active scalp electrodes (Acticap, Munich) located at standard positions (10-5 montage) via BrainAmp amplifier (BrainProduct, Munich) setup with a nose reference and ground placed at AFz. The vertical electro-oculogram (EOG) was recorded from one electrode placed to the right of the external canthus located below Fp2 electrode. Sampling rate was 500 Hz.

Continuous EEG data collected during exposure and test phases were filtered offline with a bandpass filter of 0.1–30 Hz, the slope of the filters was 24 dB/octave. In the exposure phase, eye blink artifacts were removed using Independent Component Analysis decomposition [32]. ERP data were analyzed by computing the mean amplitude, starting 100 ms before the onset of the tone and ending 1000 ms after the onset of the tone. In the test phase, epochs exceeding +/−60 µV at midline electrodes were discarded. Remaining trials with movement artifacts in both phases and with blink artifacts and/or eyes saccades in the test phase were excluded using a semi-automatic artifact-rejection procedure. ERP data were analyzed by computing the mean amplitude, starting 100 ms before the onset of the tone and ending 600 ms after the onset of the tone. Epochs were averaged for each condition (mistune/in-tune; grammatical/ungrammatical) and each participant and then averaged across participants to compare groups. Regions of interest (ROI) were defined as follow: left-frontal (F7, F5, F3, FC5, FC3), left-central (C5, C3, CCP5h, CP5, CP3), left-parietal (P7, P5, P3, PO7, PO3), right-frontal (F8, F6, F4, FC6, FC4), right-central (C6, C4, CCP6h, CP6, CP4) and right-parietal (P8, P6, P4, PO8, PO4) regions.

**Figure 1:**
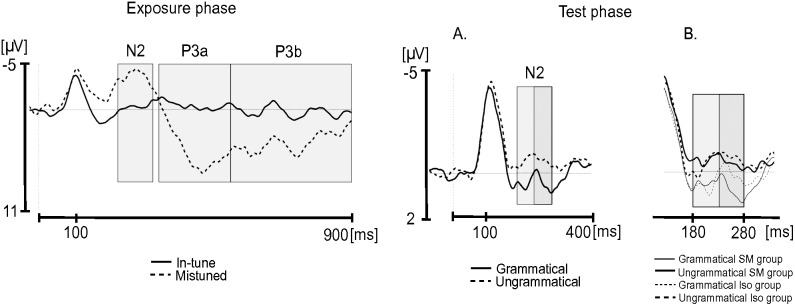
Presentation of the time windows for analyses of ERP components in the exposure phase (left) and the test phase (right) on the Cz electrode. Grey rectangles indicate time windows chosen for the analyses. Exposure phase: Time windows of N2, P3a and P3b for in-tune and mistune tones averaged over participant groups. Test phase: Subdivision of the N2 into ascending (light grey rectangle) and descending (dark grey rectangle) parts for grammatical and ungrammatical target tones averaged over participant groups (A) and separated for SM and Iso groups (B).

In the exposure phase, the mistuned tones elicited a large N2, followed by large P3a and P3b. The time windows for the analysis of the potentials amplitude were chosen according to visual inspection of grand averages (Figure 1). The N2 was defined as the mean peak amplitude within the post-stimulus time window 230–330 ms. The P3a and P3b were defined as the mean amplitudes within the post-stimulus time windows 350–550 ms and 550–900 ms, respectively. For each of these three main time windows, a repeated measure ANOVA with item type (mistuned vs. in-tune), hemisphere (left vs. right), region (frontal vs. central vs. parietal) and electrode (5 electrodes in each ROI) as within-participant factors and group as between-participants factor was performed.

In the test phase, the ungrammatical tones elicited a large N2 peaking at approximately 230 ms. The N2 was defined as the mean peak amplitude within the post-stimulus time window 180–280 ms (peak value ±50 ms) (Figure 1). Because visual inspection revealed a group difference in the ascending part of the N2, we also ran additional analyses on the ascending part and the descending part of the N2 (180–230 ms and 230–280 ms, respectively). These analyses used the same ANOVA model with item type (grammatical vs. ungrammatical), hemisphere (left vs. right), region (frontal vs. central vs. parietal) and electrodes (5 electrodes in each ROI) as within-participant factors and group as between-participants factor. All p-values reported below were adjusted using the Greenhouse-Geisser correction for nonsphericity, when appropriate, and Tukey tests were used in post-hoc comparisons. Statistical analyses were performed using Statistica software (version 10).

## Results

### Behavioral data

#### Exposure phase

The SM group detected more mistuned tones (88.57%) than did the Iso group (80.47%; *U* = 32.0, *z* = 2.475, *p* = .01, Mann-Whitney test*).* However, the two groups did not differ in their false alarm rate (21.03% and 23.45% for SM and Iso groups, respectively, *p* = .98).

#### Test phase

**Figure 2:**
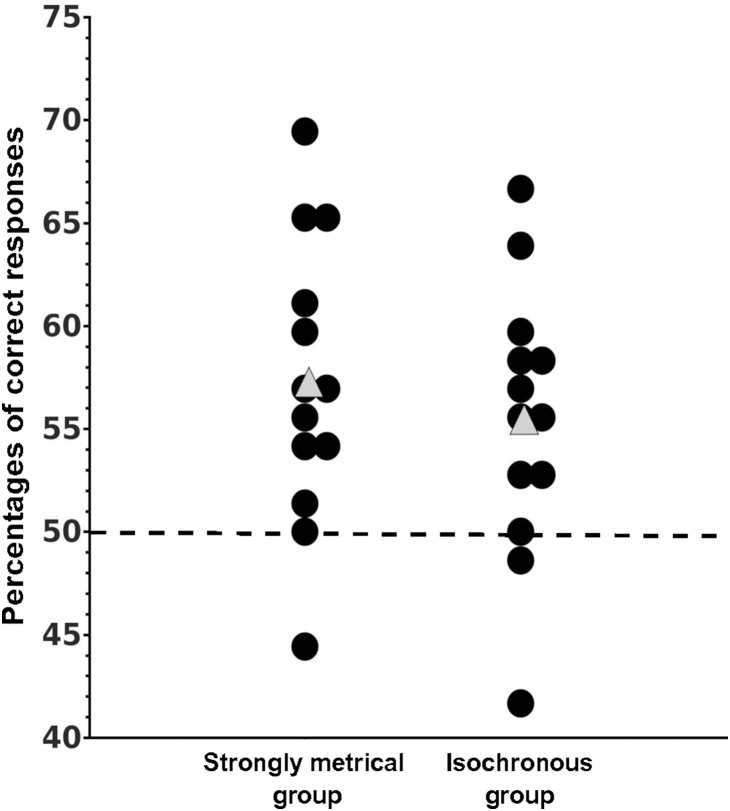
Percentages of correct responses for strongly metrical and isochronous exposure groups for each participant represented by a black dot. Grey triangles represent the average performance for each group, the dotted line represents chance level performance.

Percentages of correct responses were above chance level for participants in both groups, 55.45% (*SD* = 6.55) for the Iso group (*p = *.02, Wilcoxon test) and 57.26% (*SD* = 6.90) for the SM group (*p*<.01, Wilcoxon test) (Figure 2). Performance of the two groups did not differ significantly (*p* = .56; Mann-Whitney test).

For the confidence ratings, the ANOVA revealed a main effect of response, *F*(1,24) = 10.38, *MSE* = 0.800, *p* = .004: The confidence ratings for correct responses were higher than for incorrect responses (2.55 (*SD* = 0.79) and 2.30 (*SD* = 0.73), respectively). The main effect of group was marginally significant showing that the SM group tended to be more confident in their responses (2.68 (*SD* = 0.57) than the Iso group (2.16 (*SD* = 0.85), (*F*(1,24) = 3.61, *MSE* = 3.54, *p* = .06). The interaction between response and group was not significant (*p*>.56).

### Electrophysiological data

#### Exposure phase

**Figure 3:**
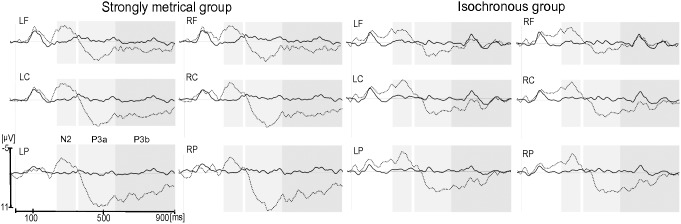
Exposure phase: Grand-average ERPs for in-tune (solid line) and mistuned (dashed line) target tones in the Strongly Metrical group (left) and Isochronous group (right). Each site represents the mean of the five electrodes included in the ROI (LF, left frontal; LC, left central; LP, left parietal; RF, right frontal; RC, right central; RP, right parietal). Grey rectangles indicate time windows of the N2 (230–330 ms), P3a (350–550 ms) and P3b (550–900 ms) chosen for the analyses.

**Figure 4:**
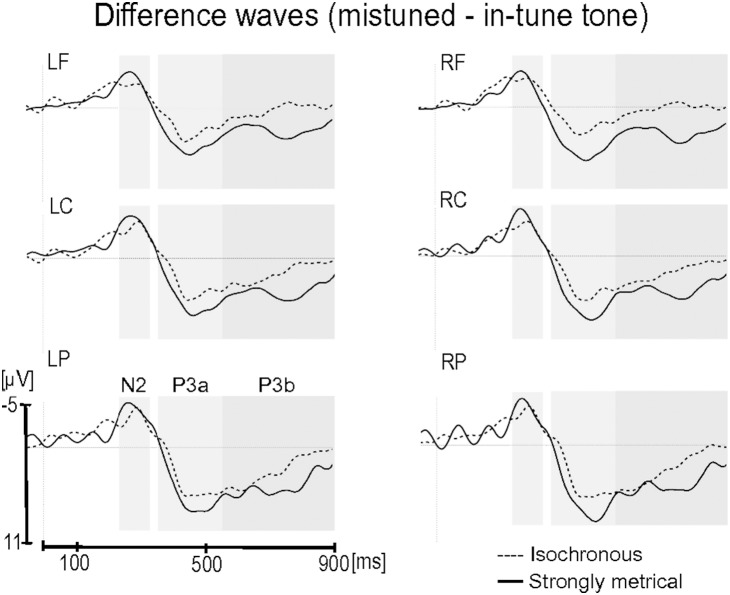
Exposure phase: Difference waves (mistuned minus in-tune target tones) for the Isochronous group (dashed line) and Strongly Metrical group (solid line). Each site represents the mean of the five electrodes included in the ROI (LF, left frontal; LC, left central; LP, left parietal; RF, right frontal; RC, right central; RP, right parietal). Only for visualization purposes waveforms are presented with a 10 Hz low-pass filter. Grey rectangles indicate time windows of the N2 (230–330 ms), P3a (350–550 ms) and P3b (550–900 ms) chosen for the analyses.

For the N2 component, the main effect of item type was significant, *F*(1,23) = 24.33, *p*<.0001, with a larger negativity for mistuned tones than for in-tune tones, (in-tune, −0.30 µV; mistuned, −3.74 µV) (Figures 3 and 4). The interaction between item type and group was not significant, *p*>.76).

For the P3a component, the main effect of item type was significant, *F*(1,23) = 28.82, *p*<.0001, with a larger positivity for mistuned tones than in-tune tones, (in-tune, −0.36 µV; mistuned, 3.75 µV). No interaction was found between item type and group, p>.13.

For the P3b component, the main effect of item type was significant, *F*(1,23) = 26.77, *p*<.0001, with a larger positivity for mistuned tones than for in-tune tones, (in-tune, −0.14 µV; mistuned, 2.68 µV). The main effect of region was also significant, *F*(2,46) = 37.68, *p*<.0001, that is larger in the parietal region in comparison to central (*p*<.001) and frontal regions (*p*<.001). Importantly, the interaction between item type and group was significant, *F*(1,23) = 5.74, *p* = .025: the mistuned tones elicited a larger positivity than did the in-tune tones, and this difference was larger for the SM group than for the Iso group (*p* = .0002 and *p* = .25, respectively; effect size (mistuned – in-tune tone): SM group, 4.14 µV; Iso group, 1.52 µV).

#### Test phase

**Figure 5:**
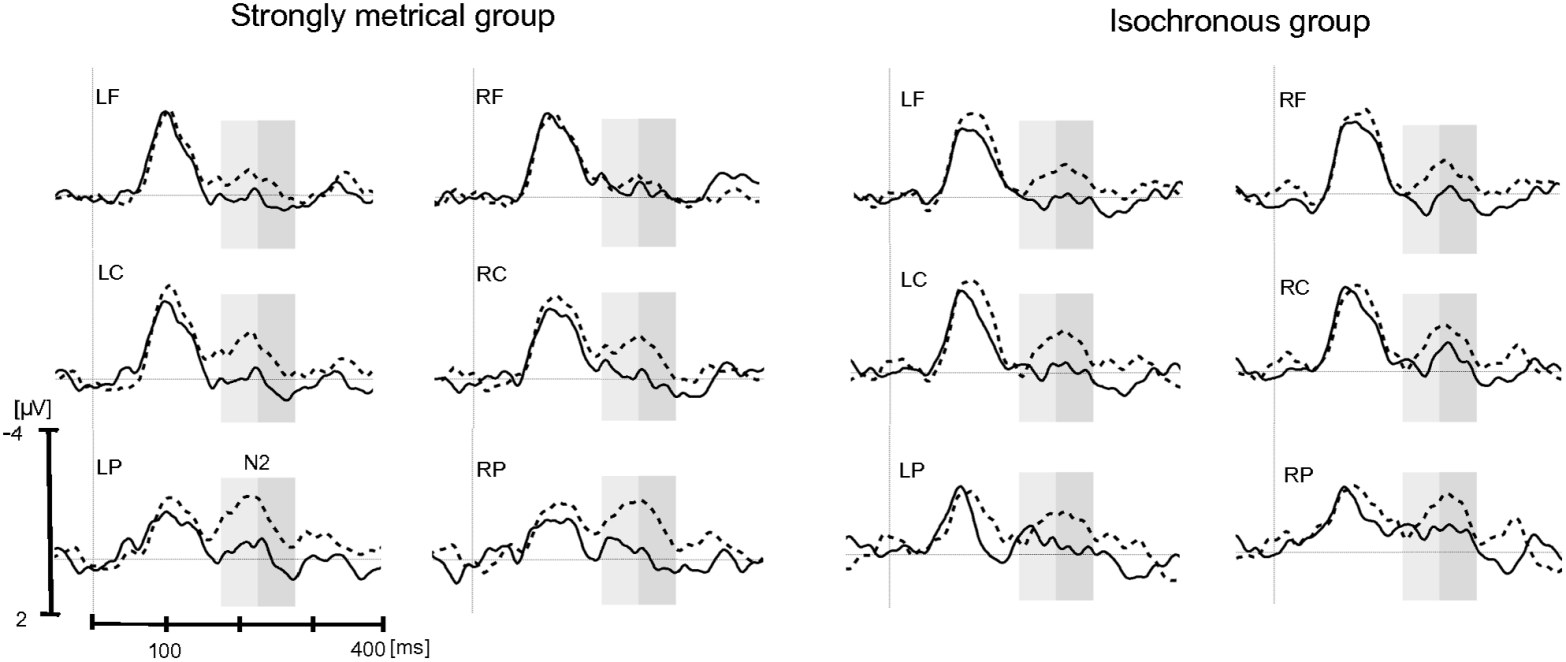
Test phase: Grand-average ERPs for grammatical (solid line) and ungrammatical (dashed line) target tones in the Strongly Metrical (left side) group and Isochronous group (right side). Each site represents the mean of the five electrodes included in the ROI (LF, left frontal; LC, left central; LP, left parietal; RF, right frontal; RC, right central; RP, right parietal). Grey rectangles indicate time windows of ascending (light grey) and descending (dark grey) part of the N2 chosen for the analyses.

**Figure 6:**
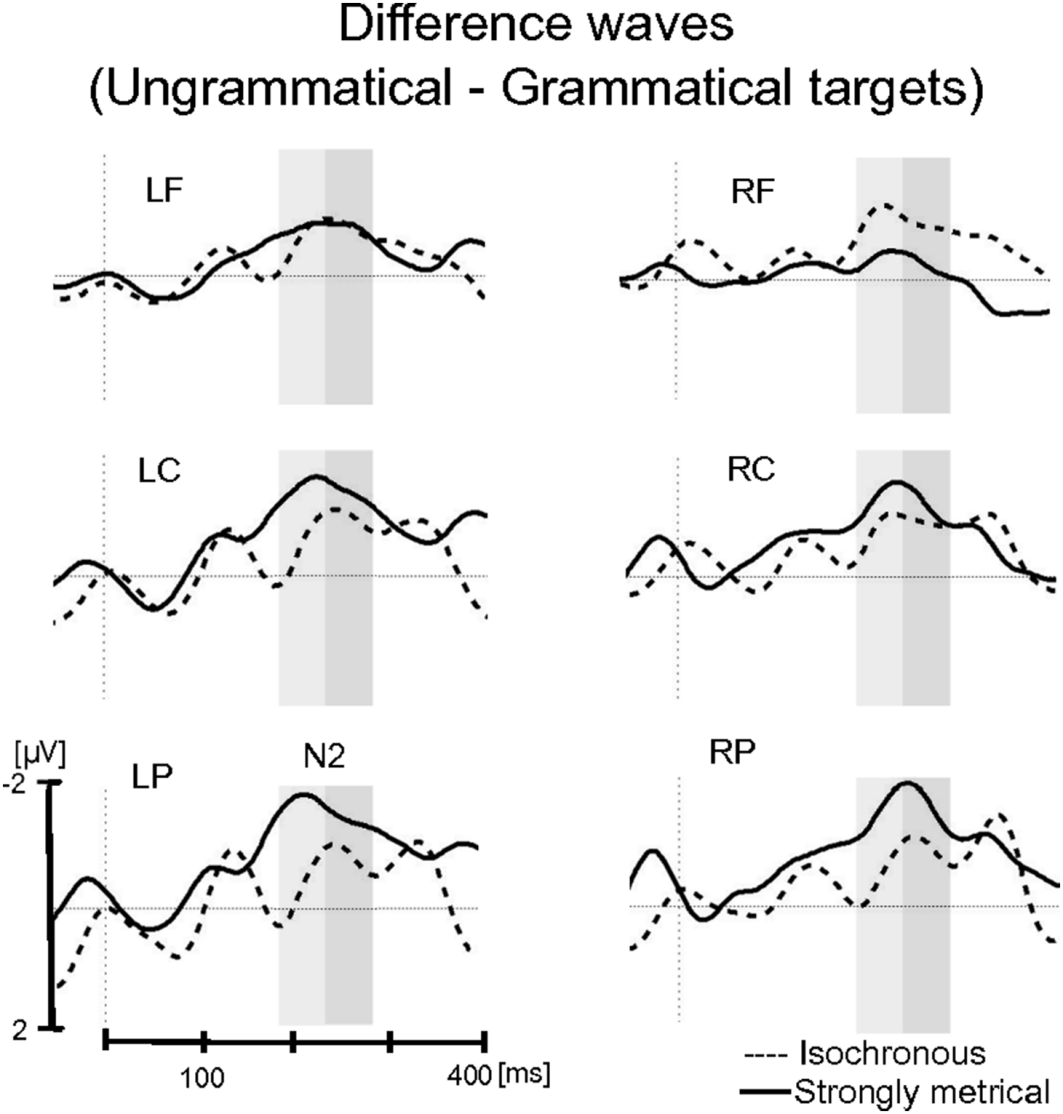
Test phase: Difference waves (ungrammatical minus grammatical target tones) for the Isochronous group (dashed line) and Strongly Metrical group (solid line). Each site represents the mean of the five electrodes included in the ROI (LF, left frontal; LC, left central; LP, left parietal; RF, right frontal; RC, right central; RP, right parietal). Only for visualization purposes waveforms are presented with a 10 Hz low-pass filter. Grey rectangles indicate time windows of ascending (light grey) and descending (dark grey) part of the N2 chosen for the analyses.

For the N2 component, the main effect of grammaticality was significant, *F*(1,24) = 11.06, *p* = .003, with a larger negativity for ungrammatical target tones than for grammatical target tones (grammatical, −0.22 µV, ungrammatical, −1.15 µV) (Figures 5 and 6). The interaction between grammaticality, group and region was significant, *F*(2,48) = 4.91, *p* = .031: The effect of grammaticality was stronger for the SM group than for the Iso group, and this was modified by region. The separate analyses of ascending (180–230 ms) and descending (230–280 ms) parts of the N2 revealed an interaction between grammaticality, group and region only for the ascending part (*F*(2,48) = 4.99, *p* = .031): While the SM group showed an effect of grammaticality over central and parietal regions (all *ps*<.003), no differences due to grammaticality were visible in the Iso group (all *ps*>.08). The analysis of the descending part revealed only a main effect of grammaticality (*F*(1,24) = 9.42, *p* = .005), with a larger negativity for ungrammatical tones than for grammatical tones. The main effect of group and the interactions involving group were not significant (*ps*>.18). Note that we also performed a statistical analysis in the N1 time window (85–125 ms), but neither the main effect of grammaticality (*p*>.42) nor the interaction between grammaticality and group (*p*>.97) were significant.

## Discussion

The present study aimed to investigate the influence of temporal structures on the learning of an artificial grammar. For this aim, we compared learning of the same artificial pitch grammar in two temporal contexts: Participants were exposed to tone sequences presented either isochronously or with rhythmic patterns that had a strongly metrical structure. Both groups learned the artificial grammar. With the behavioral measurement, no difference was found between the performance of the two groups. Interestingly, ERPs in the exposure and the test phases revealed a benefit of the strongly metrical presentation over the isochronous presentation. We first discuss the results of the test phase and then those of the exposure phase.

The grammatical violations in the test phase elicited a negativity between 180 and 280 ms for both participant groups, suggesting that participants had learned the grammatical structure of the tone sequences and detected the ungrammaticality. This result is in line with previous implicit learning studies showing that violations to newly learned sequence structures alter the N2: this has been observed in a different implementation of the AGL paradigm with verbal material [6], as well as in the SRTT [12], [16], [17], [33]. In addition, in music perception, violations of tone expectations – that is, expectations based on tonal structures learned over long term in every day life - have also been shown to affect early negativities, such as the N1 (e.g, [34]), the ERAN (early right anterior negativity) (e.g, [35]) and the right antero-temporal negativity (e.g., [36]).

In our study, the observed negativity (which could be also labelled as N2) was earlier for the SM group than for the Iso group. The N2 may represent working memory processes that are associated with template mismatch [37]. Sams et al. assumed that participants need to maintain a good mental template in order to be able to discriminate between standard and deviant stimuli, and this would be reflected by the N2. In our study, the newly acquired artificial grammar and the expectations developed on the basis of this structured system might thus represent the new mental template to which the incoming events (here tones) are compared. The N2 may indicate a mismatch between the newly acquired implicit knowledge (and notably the expectations for the next upcoming tones) and the incoming tone. Thus, the earlier N2 amplitude to grammatical violations in the SM group (in comparison to the Iso group) might suggest that the artificial pitch material was better acquired by the SM group, allowing participants to develop stronger pitch expectations and to reach an earlier mismatch with the ungrammatical event.

Our results extend previously reported data from verbal to nonverbal materials, from isochronous to complex temporal structures and to an improved test phase (i.e., use of more subtle grammatical violations and an implicit task) [6]. First, we provide evidence for the learning of nonverbal pitch material embedded in an isochronous structure as well as in more complex temporal structures (i.e., the strongly metrical presentations). Second, Carrión and Bly [6] tested unexpected events that were allover quite strong violations. In particular, they used context violations, which introduced, for example, a novel letter and grammatical violations, in which a trained letter was placed in a new position. However, even these “grammatical violations” are strong violations because they create new unseen bigrams (local violations), which can be detected without more general grammar learning. In contrast, in our study, the N2 was elicited by well-controlled, subtle grammar violations and thus suggests the acquisition of new grammar knowledge during the exposure phase. Third, in contrast to Carrión and Bly who asked participants for direct grammaticality judgments, our participants were never told about the rule-governed nature of the experimental material, that is the task remained implicit even in the test phase. Consequently, the ERP response observed for grammatical violations with this type of task might indicate that this difference in the N2 component reflects implicit knowledge acquisition. This result extends SRTT data that showed an appearance of the N2 at deviant targets in implicit learners, while a N2/P3 complex emerged in explicit learners [12], [16], [17].

In the exposure phase, we investigated how different temporal presentations may influence pitch processing, notably the processing of mistuned tones. Based on the DAT, both temporal presentations, i.e. the isochronous and strongly metrical ones, allow developing temporal expectations, but to a different extent, as suggested by the metric binding hypothesis [28]. As the isochronous pattern contains only one oscillatory level (equal to the IOI), listening to sequences with an isochronous presentation allows for the development of equal expectations for all forthcoming events (though see [38] for a different perspective). In contrast, the strongly metrical patterns incorporate at least two oscillatory levels, i.e. a lower oscillatory level with a period of 440 ms (as does the isochronous pattern) and a higher oscillatory level with a period of 880 ms. Binding these two oscillatory levels should lead to the creation of metric clusters that hierarchize and strengthen temporal expectations [28]. These stronger expectations may help listeners to better process the incoming information, and notably, here in our material, allow for better processing of the pitch dimension. Indeed, ERPs and behavioral measures of the exposure phase demonstrated better mistuned-tone processing, as reflected in increased accuracy and a larger P3 for the SM group than for the Iso group. This enhanced processing of the pitch dimension might then improve pitch structure learning as revealed by the test phase with an earlier negativity to grammatical violations for the SM group than for the Iso group.

In the exposure phase, the mistuned tones elicited a larger N2/P3 complex than the in-tune tones for both participant groups. This result is in agreement with previous findings observed for the processing of pitch deviations. In Marmel et al. [39], out-of-tune tones elicited a larger N2/P3 complex than in-tune tones. Furthermore, incongruities in pitch contour have been also reported to elicit a larger negative component that ended around 200 ms followed by a large positivity between 200 and 800 ms [40]. In our study, the difference between the two groups in mistune-tone processing was observed in the P3. Mistuned target tones elicited a significantly larger P3 in the SM group than in the Iso group. Interpreting this result according to the metric binding hypothesis suggests that the strongly metrical presentation allows guiding more precisely attention to the next expected events than does the isochronous presentation, resulting in enhanced pitch processing. Similarly, previous studies reported more effective deviant tone processing when embedded in highly predictable temporal structures, as reflected in a larger and early P3b [22], [23]. In line with the DAT, Schwartze et al. [23] suggested that this P3b effect might reflect stimulus-driven synchronization of attention that leads to facilitation of tone processing.

The parietal P3b activity may be also related to context updating operation and subsequent memory storage [41]. The findings thus suggest that working memory operations can be improved when the artificial grammar was presented strongly metrically rather than isochronously. When participants work on the mistune tone detection task, they might do real-time comparisons of the previous tones with the present tone. When listening to a melody, all previous tones are held in memory buffer in order to be compared with the next incoming tone. In line with the metric binding hypothesis, we assume that the stronger metrical presentation may help to direct attention more precisely in time (due to the two oscillatory levels binding), helping to compare more easily the current tone with the previous tones in the memory buffer. This may facilitate perceptual and cognitive processing (i.e. mistuned tone processing and implicit learning) during the exposure phase.

In sum, our present study showed that a strongly metrical presentation benefits the learning of an artificial grammar in comparison to an isochronous presentation. This facilitation might be related to better temporal processing in a subcortico-cortical network involving the basal ganglia, the frontal cortex and the cerebellum [42]. In line with the DAT, this network helps to extract temporal regularities of external events, and to generate temporal expectations, thus leading to facilitated perceptual and cognitive processes. Future studies are needed to investigate whether patients with lesions in either the basal ganglia or the inferior frontal cortex (with previously reported deficits in IL, [43], [44]) might benefit from the strongly metrical presentation and show learning of an artificial grammar.

## Supporting Information

Table S1 **Strongly metrical patterns used to create the artificial pitch sequences.**
(DOCX)

Sound example S1 **An exposure sequence with the strongly metrical presentation.**
(WAV)

Sound example S2 **An exposure sequence with the isochronous presentation.**
(WAV)

Sound example S3 **A test sequence with the strongly metrical presentation.**
(WAV)

Sound example S4 **A test sequence with the isochronous presentation.**
(WAV)

Sound example S5 **An exposure sequence with mistuned tone.**
(WAV)
