# Supplementary material for: Metrical Presentation Boosts Implicit Learning of Artificial Grammar
Source: PLoS One. 2014 Nov 5;9(11):e112233. doi: 10.1371/journal.pone.0112233 (PMC4221617; doi:10.1371/journal.pone.0112233)
Supplement: Table S1 — Strongly metrical patterns used to create the artificial pitch sequences. (DOCX) [file pone.0112233.s001.docx]

**Table S6 Strongly metrical patterns used to create the artificial pitch sequences**

*X represents a tone with duration of 220 ms, ‘.’ represents a silence of 220 ms*

|  | 10-item patterns | 12-item patterns |
| --- | --- | --- |
| 1 | X...X..XX...X..XX...X.X.X | X…X.X.X..XX…X.X.X…X.X.X |
| 2 | X...X...X.X.X..XX...X..XX | X.X.X…X..XX…X.X.X…X..XX |
| 3 | X.X.X…X.X.X…X..XX…X | X.X.X…X.X.X…X…X..XX.X.X |
| 4 | X…X..XX…X.X.X…X.X.X | X…X…X..XX.X.X..XX.X.X…X |
| 5 | X.X.X…X…X.X.X…X..XX | X…X..XX..XX…X…X.X.X..XX |
| 6 | X..XX.X.X…X…X…X..XX | X…X.X.X..XX.X.X…X..XX…X |
| 7 | X…X..XX.X.X…X..XX…X | X…X..XX…X.X.X…X.X.X.X.X |
